# Prevalence, Influencing Factors and Attitudes Towards Patient‐Led Covert Recording: A Cross‐Sectional Study of Nurses in Western China

**DOI:** 10.1002/nop2.70482

**Published:** 2026-03-09

**Authors:** Lanlan Zheng, Jie Tang, Jun Yang, Fang Zhu, Feng Peng, Hui Yang

**Affiliations:** ^1^ School of Medicine University of Electronic Science & Technology of China Chengdu China; ^2^ Nursing Department Dazhou Central Hospital Dazhou China; ^3^ Department of Otolaryngology Chengdu First People's Hospital Chengdu China; ^4^ Hospital of Chengdu University of Traditional Chinese Medicine Chengdu China; ^5^ Department of Anesthesiology The First Affiliated Hospital of Chengdu Medical College Chengdu China; ^6^ Nursing Department Sichuan Clinical Research Center for Cancer, Sichuan Cancer Hospital & Institute, Sichuan Cancer Center, University of Electronic Science and Technology of China Chengdu China

**Keywords:** attitude, coping strategies, covert recording, nurses, nursing, workplace violence

## Abstract

**Aim:**

To investigate the prevalence of patient‐led covert recording in Western China, nurses' attitudes towards covert recording and explore the factors.

**Design:**

A descriptive cross‐sectional study.

**Methods:**

This study was conducted in 33 cities in 6 provinces of China from November to December 2021. A convenience sampling strategy was used to get access to 2323 participants, and a self‐designed questionnaire was used for evaluation.

**Results:**

This study included 2124 nurses; of this, 442 (20.8%) had the experience of patient‐led covert recording. After being covertly recorded, they felt stressful (76.9%), nervous (61.3%) and angry (58.6%). A total of 1287 (60.6%) nurses didn't accept covert recording under any condition. There were differences in age, education, professional titles, years of working, departments, and management measures launched by hospitals between the groups with or without the experience of being covertly recorded. Furthermore, the binary logistic regression analysis showed that nurses running outpatient clinics (OR = 0.635, 95% CI: 0.425–0.950, *p* < 0.05) were accompanied by higher prevalence of patient‐led covert recording while the opposite was true when hospitals launched related management measures (OR = 1.632, 95% CI: 1.126–2.366, *p* < 0.05).

**Conclusions:**

Patient‐led covert recording might be a form of workplace violence which could be stressful. Thus, it deserves attention and consideration. Management measures and training on patient‐led covert recording are urgently required to meet the needs of nurses.

**Patient or Public Contribution:**

The findings of this study emphasise the significant role of patient and public contributions in healthcare dynamics. By revealing nurses' stress and anxiety associated with covert recordings, it highlights the need for open dialogue about privacy and consent. Engaging patients in discussions about the ethical implications of such recordings can foster collaboration and trust in clinical settings. The findings suggest that implementing better management measures in hospitals could alleviate negative impacts and enhance communication between patients and healthcare providers.

**Trial Registration:**

Chinese Clinical Trial Registry: ChiCTR2100048557

## 1 Introduction

In the era of self‐media, with the widespread use of smartphones and habit of casually recording, covert recording are not an uncommon occurrence (Elwyn et al. 2017; Hampton and Constantz 2024; ‘How to Legally Limit Recording Devices in the Workplace’ 2018; Paine 2021; Rodriguez et al. 2015). In the healthcare industry, nurses are the most likely to be recorded by patients as they have the most contact with them. Patient‐led covert recording happens when patients record medical personnels without knowledge or consent of them (Prictor et al. 2021; Ryan et al. 2022a). However, being recorded by patients without awareness may result in damage to the relationship between patients and nurses. As a global issue, patient‐led covert recording has attracted increasing attention. Elwyn et al. (2015) carried out a qualitative and quantitative research on the motivations of recording, involving 168 patients and reported that 19 (15%) respondents had covertly recorded a clinical encounter and 45 (35%) of them said they would covertly record; In total, 69% of respondents indicated the desire to record clinical encounters. Barr et al. (2018) investigated 524 public respondents and reported that 14 (2.7%) of them made audio or video recording without permission. It may because some healthcare institutions routinely offer patients recordings of their clinic visits. A survey of oncologists showed that 333 (93%) of them had experienced recording during a clinic visit, with 47 (14%) of them rarely or never were asked permission and 190 (57%) of them reported experiencing recording with permission (Jimenez et al. 2022).

Despite the high prevalence of covert recording among medical personnel, there is still no unanimity on this issue (Paine 2021). It is mainly because recording has different influences on patients and medical personnel, including enhancing patient and caregiver's understanding of encounters, improving their education, recalling and understanding information, improving patients' engagement and satisfaction, as well as raising concerns about recording a low‐quality service among medical personnel (Barr et al. 2018; Elwyn et al. 2015, 2017; Jimenez et al. 2022; Ryan et al. 2023; Ryan, Weir, Maskell, and Le Brocque 2022; Tsulukidze et al. 2014; Turley and Metcalfe 2020). Although most physicians believed that recording could positively impact the patients, there was still a greater diversity of views about its influence on the patient‐physician relationship (Jimenez et al. 2022). Thus, institutional policies about recording need to weigh the benefits and drawbacks of this behaviour.

In the health‐care system, nurses are given a frontline position. Thus, it is of vital importance to understand their attitudes towards patient‐led covert recording and how it affects them because they may encounter this situation during their work. However, there are a limited number of studies related to the prevalence and influencing factors of patient‐led covert recording and nurses' attitudes towards it. What's more, appropriate coping strategies are needed to develop. Therefore, this study aims to investigate the factors affecting patient‐led covert recording, in order to provide reference for properly coping with patient‐led covert recording.

## 2 Methods

### 2.1 Study Design and Participants

A descriptive cross‐sectional design was used and registered on July 10, 2021, including fulfilling a self‐reported questionnaire which took about 2 min. This study was carried out from November to December 2021. By using convenience sampling, the questionnaires were passed on to nurses by the director of the hospital management department. Eligible participants were registered nurses currently providing direct patient care in clinical departments. Nurses in purely administrative, research, or educational roles were excluded. 2323 nurses were involved in this study from 33 cities in 6 provinces of China, and 2124 valid questionnaires were included in the final analysis.

### 2.2 Data Collections

The questionnaire was released by Survey Star platform. To get access to the questionnaire, the participant was required to click the link address or scan QR code via WeChat. Most of the questions were set to a single choice and they may have multiple choices in certain questions. Each of the questions was required to be completed before submission, while participants without the experience of being covertly recorded or didn't know whether they had been covertly recorded were not required to fulfil the extra questions related to patient‐led covert recording experience. Invalid questionnaires were identified based on the following criteria: (a) patterned or identical responses throughout; (b) an implausibly short completion time (< 60 s); or (c) missing data for > 10% of the items. A total of 199 responses were removed based on these quality checks.

### 2.3 Instrument

A self‐administered questionnaire of patient‐led covert recording experience for nurses was developed via a systematic literature review and expert consultation. This questionnaire was divided into three parts, including (1) sociodemographic characteristics, (2) participants' perceptions towards covert recording (12 items) and (3) the characteristics of exposure to covert recording (7 items), two dimensions, 19 items.

#### 2.3.1 Socio‐Demographic Characteristics

Socio‐demographic data included gender, age, education, professional title, years of working, hospital accreditation level and department.

#### 2.3.2 Participants' Perceptions Towards Patient‐Led Covert Recording

This part investigated the prevalence of patient‐led covert recording and demonstrated participants' knowledge, attitudes and practices regarding covert recording, including the legality and acceptance of covert recording, reasons for rejecting covert recording, acceptable conditions for covert recording, reasons for covert recording from nurses' perspective, whether or not they had received training and management measures about covert recording, desired training formats to respond to covert recording, whether or not to covertly record others.

#### 2.3.3 Characteristics of Exposure to Patient‐Led Covert Recording

This part included the places that covert recording happened, ways to discover covert recording, nurses' feelings of being covertly recorded, coping strategies and reasons for covert recording from patients' perspectives.

A two‐round Delphi consultation was conducted with a panel of nine senior nursing managers. In the first round, experts rated the relevance and clarity of each proposed item and provided open‐ended feedback. Items with an Item‐Content Validity Index (I‐CVI) below 0.78 were revised or removed, and the wording of several items was clarified based on expert suggestions to better fit the clinical nursing context. The revised questionnaire was re‐evaluated in the second round, achieving a final Scale‐Content Validity Index (S‐CVI) of 0.93. The questionnaire comprises multiple question formats designed to capture the multidimensional nature of the phenomenon. As this was a newly developed instrument and a formal pilot test was not conducted prior to the main survey, traditional reliability coefficients are not reported.

### 2.4 Data Analysis

The statistical analysis was performed with the programme IBM SPSS Statistics version 20.0. The demographic characteristics and work context of participants, including age, gender, educational level, professional qualifications, hospital accreditation level, department, years of working as well as their attitudes towards patient‐led covert recording were analysed by descriptive statistics. Continuous variables were represented by mean and standard deviations and categorical variables by frequencies and percentages. Participants were stratified into two groups by experience of being covertly recorded. One‐way analysis of variance is used to identify the difference between groups. Chi‐square tests were performed to examine associations between categorical demographic or professional variables and the binary outcome of experiencing covert recording. These tests assessed whether the proportion of nurses reporting such experience differed across subgroups, even against the background of an overall lower prevalence. For these comparative analyses, only participants who provided a definitive ‘Yes’ or ‘No’ response regarding their experience of covert recording were included. Respondents who were ‘Uncertain’ about their experience were excluded from these analyses due to the indeterminate nature of their outcome status. For the binary logistic regression analysis, the dependent variable was the experience of patient‐led covert recording, coded as 0 = no (had not experienced) and 1 = yes (had experienced).

### 2.5 Ethical Considerations

This study was approved by the Ethics Committee before data collection. Informed consent was obtained online; participation was voluntary and anonymous. To mitigate any potential concern of coercion from the hospital‐mediated distribution, the study was explicitly presented as unrelated to job performance, and researcher contact was provided. Participants' information was kept confidential, and data were used solely for this study.

## 3 Results

### 3.1 Participants Characteristics

In total, 2323 nurses participated in this study, and 2124 nurses' data were valid (91.4%) and included in the final analysis. Most participants (98.1%) were female. The average age of the participants was 32.07 ± 7.89 years, with an average of 10.47 ± 8.40 years of work experience. In terms of highest education level, 63.6% of participants were bachelors and only 1.3% held a Master's degree or above. Most nurses (63.4%) held junior titles. More than half of participants worked in Grade III hospitals. More than half of them worked in inpatient departments, and 7.5% of the participants ran outpatient clinics (Table 1). The 2124 participants worked in hospitals located in 33 cities of 6 provinces in Western China.

**TABLE 1:** Demographic and professional characteristics of study participants (*n* = 2124).

Items	Categories	Frequency (%)
Gender	Male	41 (1.9)
	Female	2083 (98.1)
Age	< 25	325 (15.3)
	25–34	1135 (53.4)
	35–44	471 (22.2)
	45–54	166 (7.8)
	≥ 55	27 (1.3)
Highest educational level	Technical secondary school	49 (2.3)
	Junior college	697 (32.8)
	Bachelor	1350 (63.6)
	Master or above	28 (1.3)
Professional title	Junior	1346 (63.4)
	Intermediate	571 (26.9)
	Deputy senior	175 (8.2)
	Senior	32 (1.5)
Years of working	< 5	569 (26.8)
	5–9	590 (27.8)
	10–19	646 (30.4)
	20–29	208 (9.8)
	≥ 30	110 (5.2)
Hospital accreditation level	Grade III‐A	899 (42.3)
	Grade III‐B	278 (13.1)
	Grade II‐A	800 (37.7)
	Grade II‐B	147 (6.9)
Department	Inpatient department	1277 (60.1)
	Outpatient and technological departments	847 (39.9)
Whether or not to run outpatient clinic	Yes	159 (7.5)
	No	1965 (92.5)

### 3.2 Overall Status of Nurses' Perceptions and Attitudes Towards Patient‐Led Covert Recording

Of the 2124 nurses investigated, 442 (20.8%) had experienced patient‐led covert recording at least once and 1436 (67.6%) of participants considered covert recording illegal. What's more, 1897 (89.3%) of nurses couldn't accept this behaviour, with the major reason that it interfered with their working state. However, 985 (46.4%) of nurses could accept this behaviour only if patients used the recording for home health education. As for the reasons for covert recording from nurses' perspective, 1888 (88.9%) of them thought it was patients' mistrust of medical personnel, followed by too much negative press in the social media (77.4%) and patients' thirst for knowledge of diseases (59.1%) that led to patient‐led covert recording.

The hospitals where 1690 (79.6%) participants worked in didn't launch the management measures for covert recording, 1837(86.5%) of them didn't receive any training about covert recording and their most desired training format was case analysis (78.7%). Of 1945 (91.6%) nurses would not covertly record the work of other medical personnels when they acted as a patient or patient's family member, and 2035 (95.8%) of nurses never covertly recorded the working scenes of staffs in other industries. Table 2 shows the nurses' perceptions and attitudes towards covert recording.

**TABLE 2:** Study participants' perceptions and attitudes about patient‐led covert recording (*n* = 2124).

Items	Categories	Frequency (%)
Covertly recorded by patients	Yes	442 (20.8)
	No	968 (45.6)
	Uncertainty	714 (33.6)
Whether covert recording is legal	Yes	149 (7.0)
	No	1436 (67.6)
	Uncertainty	539 (25.4)
Acceptance of covert recording	Yes	111 (5.2)
	No	1897 (89.3)
	Indifferent attitude	116 (5.5)
Reasons for rejecting covert recording	Interfering with working state	1787 (84.1)
	Breach of legality	1631 (76.8)
	Ethics violations	1418 (66.8)
	Negative affect on personal feelings	1284 (60.5)
	Others	612 (28.8)
Acceptable conditions for covert recording	No circumstance is acceptable	1287 (60.6)
	Home health education	985 (46.4)
	Record life	603 (28.4)
	Share with friends and family	210 (9.9)
Reasons for covert recording from nurses' perspective	Patients mistrust Medical personnels	1888 (88.9)
	Too much negative press in the media	1643 (77.4)
	Patients' thirst for knowledge of diseases	1256 (59.1)
	Habitual recording	802 (37.8)
	Others	386 (18.2)
Whether or not to launch management measures	Yes	434 (20.4)
	No	1690 (79.6)
Receiving training or not	Yes	287 (13.5)
	No	1837 (86.5)
Desired training format	Case analysis	1672 (78.7)
	Scenario walkthroughs	1479 (69.6)
	Theoretical lectures	1360 (64.0)
	Oral preaching	1227 (57.8)
	Others	335 (15.8)
Whether or not to covertly record the work of medical personnels	Yes	30 (1.4)
	No	1945 (91.6)
	Uncertainty	149 (7.0)
Whether or not to covertly record the work scenes of people in other industries	Yes	89 (4.2)
	No	2035 (95.8)

### 3.3 Characteristics of the Occurrence of Patient‐Led Covert Recording

In our study, 368 (83.3%) of nurses experienced patient‐led covert recording in inpatient departments, most of them discovered it by themselves (84.2%). After being covertly recorded, they felt stressful (76.9%), nervous (61.3%) and angry (58.6%). For the coping strategies, 413 (93.4%) of nurses immediately rejected this behaviour after realising it and just 14 (3.2%) nurses permitted them to continue recording. A total of 211 (47.7%) of patients covertly recorded nurses for sharing with their friends and family, and 170 (38.5%) of them aimed to obtain verifiable evidence for potential complaint procedures. Table 3 shows the nurses' reaction to covert recording and patients' reasons for it.

**TABLE 3:** Nurses' exposure to and characteristics of patient‐led covert recording (*n* = 442).

Items	Categories	Frequency (%)
Places that covert recording happened	Inpatient department	368 (83.3)
	Outpatient and technological departments	206 (46.6)
Ways to discover covert recording	By themselves	372 (84.2)
	Patients	29 (6.6)
	Informed by others	217 (49.1)
Feelings of covert recording by patients	Stressful	340 (76.9)
	Nervous	271 (61.3)
	Angry	259 (58.6)
	Humiliated	111 (25.1)
	Generate departure impulse	39 (8.8)
	No feeling	21 (4.8)
Coping strategies	Reject	413 (93.4)
	Seek hospital communication	51 (11.5)
	Languages conflicts	18 (4.1)
	Permitted	14 (3.2)
	Indifferent attitude	14 (3.2)
	Physical altercations	2 (0.5)
	Others	56 (12.7)
Patients' reasons for covert recording	Share with friends and family	211 (47.7)
	Obtain verifiable evidence	170 (38.5)
	Publish online public opinion	167 (37.8)
	Record life	150 (33.9)
	Home health education	93 (21.1)

### 3.4 The Influence of Socio‐Demographic Variables on Being Covertly Recorded

Table 4 presents the prevalence of covert recording experience across different sociodemographic and professional characteristics. The proportion of nurses who reported having been covertly recorded appeared higher among those who were aged 35–44, held a bachelor's degree or higher, had an intermediate professional title, had 5–19 years of work experience, worked in inpatient departments, ran outpatient clinics, and worked in hospitals without specific management measures against covert recording (*p* < 0.05). The prevalence did not show statistically significant variation by gender (*p* = 0.659) or hospital accreditation level (*p* = 0.086).

**TABLE 4:** Univariate analysis of the influencing factors of patient‐led covert recording that nurses suffered (*n* = 1410).

Variables	Categories	Nurses with experience, *n* = 450 (%)	Nurses without experience, *n* = 993 (%)	*χ* ^2^ value	*p*
Gender	Male	8 (27.6)	21 (72.4)	0.195	0.659
	Female	434 (31.4)	947 (68.6)		
Age	< 25	42 (20.8)	160 (79.2)	17.847	0.001
	25–34	242 (32.0)	515 (68.0)		
	35–44	119 (37.9)	195 (62.1)		
	45–54	32 (27.4)	85 (72.6)		
	≥ 55	7 (35.0)	13 (65.0)		
Highest educational level	Technical secondary school	10 (30.3)	23 (69.7)	21.736	0.000
	Junior college	107 (23.7)	345 (76.3)		
	Bachelor	313 (34.7)	589 (65.3)		
	Master or above	12 (52.2)	11 (47.8)		
Professional title	Junior	246 (28.1)	629 (71.9)	12.164	0.007
	Intermediate	148 (37.8)	244 (62.2)		
	Deputy senior	39 (34.2)	75 (65.8)		
	Senior	9 (31.0)	20 (69.0)		
Years of working	< 5	83 (22.7)	283 (77.3)	20.691	0.000
	5–9	134 (34.4)	256 (65.6)		
	10–19	162 (36.7)	280 (63.3)		
	20–29	38 (28.6)	95 (71.4)		
	≥ 30	25 (32.1)	53 (67.9)		
Hospital accreditation level	Grade III‐A	213 (34.4)	407 (65.6)	6.599	0.086
	Grade III‐B	56 (33.3)	112 (66.7)		
	Grade II‐A	148 (28.0)	380 (72.0)		
	Grade II‐B	25 (26.6)	69 (73.4)		
Department	Inpatient department	294 (34.3)	562 (65.7)	9.101	0.003
	Outpatient and technological departments	148 (26.7)	406 (73.3)		
Whether or not to run outpatient clinic	Yes	50 (41.0)	72 (59.0)	5.762	0.016
	No	392 (30.4)	896 (69.6)		
Whether or not to launch management measures	Yes	81 (25.8)	233 (74.2)	5.785	0.016
	No	361 (32.9)	735 (67.1)		

*Note:* Data are *n* (%). Comparison of two groups based on socio‐demographic and working characteristics. Statistical significance was evaluated by the Chi‐square analysis of variance test.

### 3.5 The Correlation Between Socio‐Demographic and Patient‐Led Covert Recording

The results of the binary logistic regression analysis are presented in Table 5. Among the demographic variables examined (gender, age, education level, professional title), none showed a statistically significant association with the experience of covert recording (all *p* > 0.05). Running outpatient clinic was positively correlated with covert recording experience (OR = 0.635, 95% CI: 0.425–0.950, *p* < 0.05). Management measures were negatively correlated with covert recording experience (OR = 1.632, 95% CI: 1.126–2.366, *p* < 0.05).

**TABLE 5:** Binary logistic regression model on exposure to patient‐led covert recording.

Variables	Group	*β*	*S* $\bar{x}$	Wald *χ* ^2^	*p*	OR	95% CI
Gender	Male^a^						
	Female	0.180	0.441	0.167	0.683	1.197	0.505–2.839
Age	< 25^a^						
	25–34	0.147	0.243	0.367	0.544	1.159	0.720–1.866
	35–44	0.347	0.312	1.232	0.267	1.414	0.767–2.608
	45–54	−0.054	0.478	0.013	0.910	0.948	0.372–2.416
	≥ 55	0.285	0.709	0.162	0.687	1.330	0.331–5.336
Highest educational level	Technical secondary school^a^						
	Junior college	−0.474	0.419	1.281	0.258	0.623	0.274–1.415
	Bachelor	−0.202	0.428	0.222	0.637	0.817	0.353–1.890
	Master or above	0.541	0.632	0.732	0.392	1.718	0.497–5.932
Professional title	Junior^a^						
	Intermediate	0.219	0.183	1.423	0.233	1.245	0.869–1.783
	Deputy senior	0.225	0.327	0.474	0.491	1.252	0.660–2.376
	Senior	−0.121	0.552	0.048	0.826	0.886	0.300–2.611
Whether or not to run outpatient clinic	Yes^a^						
	No	−0.453	0.205	4.894	0.027	0.635	0.425–0.950
Whether or not to launch management measures	Yes^a^						
	No	0.490	0.190	6.677	0.010	1.632	1.126–2.366

^a^ Control group.

## 4 Discussion

### 4.1 Patient‐Led Covert Recording Is a Pressing Issue for Nurses

This study not simply explores the prevalence of patient‐led covert recording encountered by nurses in Western China but also illustrates patients' motivations for this behaviour. As the results showed, 20.8% of participants had ever been covertly recorded by patients. The prevalence was higher than a mixed‐methods study related to patient recording clinical encounters in the UK (Elwyn et al. 2015) and Barr's study in five Alliance for Clinical Trials in Oncology sites across the United States (Barr et al. 2018). The possible reason is that the participants of this study were from secondary or tertiary hospitals with high patient volume and greater accessibility of recording devices. And Oyedokun's survey showed that 35.3% (42/119) of nurses had experienced a patient who wanted to record the procedures (Oyedokun et al. 2019). That means patient recording is not an uncommon phenomenon.

This study revealed that the number of participants who rejected covert recording far exceeded acceptances. Interfering nurses' working state (84.1%, 1787/2124) was the major reason for rejecting, followed by breach of legality (76.8%, 1631/2124), ethics violations (66.8%, 1418/2124) and then negative affect on personnel feelings (60.5%, 1284/2124). Among the nurses experienced covert recording in this study, feeling stressful was the most frequent emotional reaction, followed by nervous, angry, humiliated and then generated departure impulse. These findings about medical personnel's attitudes and reaction towards recording converge with, but also differ from, those of other research (Jimenez et al. 2022; McConnell et al. 1999; Ryan, Weir, Maskell, and Le Brocque 2022). In terms of broad disapproval, our finding is consistent with McConnell's study (McConnell et al. 1999), where 72.6% of GPs opposed the provision of an audiotape. However, our data provide a more granular view specific to nurses, identifying ‘interference with work’ as the paramount concern, which differs from the emphasis often placed on legal or ethical issues first in studies involving physicians. Jimenez's study found that clinicians with recording experience and reported discomfort most frequently concerns about legal liability, feeling less natural during discussion and finding recording distracting (Jimenez et al. 2022). While our participants also expressed strong concerns about legality and privacy, the predominant and most severe emotional reaction we documented was ‘stress’, which was frequently linked to a loss of control and the fear of recordings being used out of context for complaints or public dissemination. This suggests that for nurses, the immediate psychological impact and perceived threat to professional autonomy may be more acute than the abstract legal concerns highlighted in some physician‐focused studies. Covert recording raised medical personnels' concerns about medico‐legal issue, including privacy such as confidentiality and portraiture right. As reported in a qualitative analysis of online text, many clinicians deeded covert recording as a violation of their privacy and impeded the open conversation (Elwyn et al. 2015; Tsulukidze et al. 2015). The reason for feeling stressful might because nurses were concerned about patients owning evidence of mistakes or clips of their dissatisfaction with the service (Ryan, Weir, Maskell, and Le Brocque 2022). Especially when patients covertly recorded the nurses, the mistakes or prejudicial editing might be recorded and shared, which made nurses feel stressful. It is accountability that added a bit more pressure on them. Patients might just focus on the wrong parts of the recording and ignore the real issue, which made recording “risky” (McConnell et al. 1999). They may also use recordings as a basis for complaints or legal claims (Elwyn et al. 2017). What's more, loss of control and its dissemination to social media or news outlets triggered feelings of worry (Ryan, Weir, Maskell, and Le Brocque 2022). In synthesising these points, our study illuminates a specific stress pathway for nurses: the combination of direct accountability, vulnerability to selective editing, and the potential for public exposure via social media appears to converge into a profound sense of professional and personal vulnerability. This goes beyond the general privacy and legal concerns shared across professions and points to the heightened emotional labour and perceived risk inherent in the nursing role when faced with covert recording.

In this study, we identified that nurses perceived patient mistrust as the primary reason for covert recording (88.9%). This finding corroborates the observations of Tsulukidze et al. (2015), who suggested that such recording may stem from patients' experiences of perceived poor care or inadequate attention. To interpret this prevalent perception of mistrust, we consider it within the framework of workplace incivility. The act of covert recording can itself be viewed as a form of patient incivility, indicative of a ‘lack of respect’ in the clinical interaction. This perspective is significant because incivility from patients has been shown to negatively impact nurses' mental health and professional quality of life, leading to anxiety and depression (Nazari et al. 2023). Furthermore, our finding points to a potential systemic issue, as research indicates that patient incivility is negatively associated with a supportive organisational culture (Alquwez 2023). This suggests that an environment perceived as lacking in organisational support may inadvertently foster uncivil behaviours, such as covert recording. Therefore, improving organisational support and fostering a culture of respect may be crucial not only for enhancing nurse well‐being but also for mitigating the underlying dynamics that lead to patient behaviours like covert recording. In general, these insights into the perceived drivers and broader contextual factors reinforce that nurses in this study held strongly negative attitudes towards patient‐led covert recording.

### 4.2 Factors for the Incidence of Patient‐Led Covert Recording Are Complex

The following nurses' characteristics were examined in relation to patient‐led covert recording: gender, age, education, professional title, years of working, department, and whether or not to run an outpatient clinic. Univariate analysis showed that age, the education level, professional title, years of working, department, whether or not to run an outpatient clinic, and whether or not the hospitals had launched management measures affected the incidence of patient‐led covert recording, indicating that nurses who worked in the inpatient department with a bachelor's or master's degree, intermediate title, 10–19 service years, and also ran an outpatient clinic had a higher incidence of covert recording.

In contrast to previous studies (Hamdan and Abu Hamra 2015; Kitaneh and Hamdan 2012), this study showed that nurses aged 35–44 with 10–19 service years were associated with a greater likelihood of being covertly recorded than younger and less experienced nurses, which was generally consistent with Jimenez's study (Jimenez et al. 2022). It might be because the role incumbent is improved with the accumulation of work experience, which also brought them greater responsibility and a heavier workload in clinical work (Wei et al. 2019). What's more, patients tend to look for high‐level medical care (Gan et al. 2018). Those nurses with bachelor's, master's or above degree had richer specialist knowledge (Gan et al. 2018; Tian et al. 2020). As the finding of this study showed, one of the reasons for covert recording from the patient's perspective was for home health education (Gan et al. 2018). Thus, nurses with a higher education level had a higher incidence of being covertly recorded than nurses with a lower education level, which might be explained exactly by their capacity to meet patients' need for home health education. As for nurses with intermediate titles having a higher incidence of being covertly recorded than nurses with lower job titles, it might be because they took on more clinical work and responsibilities.

In this study, the binary logistic regression analysis showed that nurses running outpatient clinics were more likely to experience covert recording. This finding was paralleled to previous research that nurses who needed to run outpatient clinics had more contact with patients, which increased the likelihood of encountering covert recording (Fu et al. 2021; Tian et al. 2020). In addition, nurses who are qualified to run outpatient clinics are usually specialist nurses. The nursing role in Nurse Led Clinics involves offering patients health‐related education (Randall et al. 2017). Thus, patients might record the clinic visits to recall and improve understanding of medical information (Barr et al. 2018). Additionally, nurses working in hospitals without management measures for covert recording were more likely to be covertly recorded in this study. This finding corresponds with a study in multiple countries where many individuals believed that taking action to implement rules and regulations such as posting a sign that says recording is strictly prohibited could reduce the prevalence of patient‐led covert recording (Tsulukidze et al. 2015). The possible reason is that the existence of clear policies and regulations in hospitals to prohibit covert recording, as well as the corresponding penalties for violators, can help deter potential breaches.

However, the results of binary logistic regression analysis revealed that age, education, professional title, years of working, and department did not influence the incidence of covert recording, suggesting that further studies are needed in the future.

### 4.3 Coping Strategies of Patient‐Led Covert Recording Are Insufficient

As this study showed, nearly 90% of nurses considered covert recording a sign of mistrust. While mistrust in the workplace belongs to a form of abuse, which is the behaviour that humiliates, degrades or otherwise indicates a lack of respect for the dignity and worth of an individual (World Health Organization [WHO] 2002). And abuse is one of the most frequently used terms related to workplace violence. Therefore, patient‐led covert recording may be a form of workplace violence for most of the nurses in our study.

This study indicated that 85.1% (376/442) and 81.7% (361/442) of nurses who had experienced patient‐led covert recording didn't receive any training about how to deal with covert recording and reported no relevant regulatory measures on covert recording in their hospitals, respectively. Moreover, nurses from hospitals with covert recording‐related management were less likely to be covertly recorded. These findings highlight that hospital administrators should attach importance to regulating related management to address patient‐led covert recording, and also suggest that hospital managers urgently establish provisions for covert recording. The limited supply of management and training might contribute to their negative reaction; for example, 8.8% (39/442) of nurses who were covertly recorded in our study generated departure impulse (Elwyn et al. 2017; Rodriguez et al. 2015; Ryan, Weir, Maskell, and Le Brocque 2022).

Training to help nurses effectively decide whether to accept or refuse recording is also imperative, as it helps them take appropriate action following such events. The findings revealed that case analysis (78.7%, 1672) was the most desired training method for the nurses in this study, followed by scenario walkthrough (69.6%, 1479). Simulation‐based education for teaching management skills to nurses in hospitals can offer them an active role in a live, interactive scenario, delivering active educational experiences to participants (Mitchell et al. 2024). Therefore, hospitals could take it into account and consider nurses' wishes when developing training programmes.

What's more, the reporting system is equally important because it helps to estimate the true extent of patient‐led covert recording and provide a full spectrum of it, which is critical to indicating the need for prevention (Arnetz et al. 2015; Salarvand et al. 2024). It might also help improve nurses' physical and mental health. Thus, hospital managers could also develop a reporting system to collect accurate data from patient‐led covert recordings.

### 4.4 Legislative Issues of Patient‐Led Covert Recording Are Controversial

Covert recording is generally driven by pernicious motives (Iserson et al. 2019) and it poses significant risks to the confidentiality and privacy of both patients and medical personnel (American College of Emergency Physicians [ACEP] 2017). However, patients were unclear about the legality of recordings, especially if done covertly. In this study, about one‐quarter (539/2124) of participants were unsure of the legality of covert recording. It may be attributed to the lack of clear legal provisions in China.

In addition, laws permitting patient‐led recording vary across countries (Iserson et al. 2019). The legality of patient‐led recording is complex in the United States, where wiretapping laws differ across all‐party and single‐party jurisdictions (Elwyn et al. 2017; Smith 2016). In all‐party jurisdictions, covert recording is illegal, while in single‐party jurisdictions, patient is legal to record a clinical encounter. In the latter situation, medical personnel can choose to terminate the visit, and patients are required to obtain the agreement of those who were recorded before sharing the recordings on social media. In addition, recordings obtained in violation of the law may not be introduced as evidence in court. In Australia, recording is legislated according to the state. It's legal for patients to record the conversation without the consent of other parties if they are part of it apart from South Australia and Western Australia, where two‐party consent is required, and they need to obtain the consent of others if share it with anyone else (Prictor et al. 2021; Ryan, Weir, Maskell, and Le Brocque 2022). In the UK, recording entirely made for personal reasons is exempt from data protection principles, but medical personnels are entitled to point out that a recording without their agreement will engage their privacy rights, and the post of recording without their consent falls outside the personal use exceptions of the Data Protection Act (Data Protection 2024; Patients Recording Consultations 2024; Participation 1998).

Obtaining consent when recording is morally right or the courteous course of behaviour (Ryan et al. 2022b). Covert recording may be a breach of trust and an invasion of privacy. Thus, the most essential strategy for all medical personnel is embracing the possibility that they may be recorded by patients at any time and exploring patients' reasons when medical personnel suspect they are covertly recording (Patients Recording Consultations 2024; Rodriguez et al. 2015). Policy makers are also required to develop proactive, clear policies on patient‐led recording and actively bridge the gap between the lack of and the need for laws related to covert recording (Jimenez et al. 2022). In addition, institutional policy about the clarity requirements of recording is also urgently needed. Parallel to previous research, this study showed that 47.7% (211/424) of nurses experienced patient‐led covert recording for the major reason that patients wanted to share their treatment experience or consultation with friends and family members (Elwyn et al. 2015; Jimenez et al. 2022). Thus, policymakers should also take patients' needs into account when developing related rules and policies. The policy should clearly define the circumstances under which recording is permitted and instruct the ways in which recordings are used (Ryan, Weir, Maskell, and Le Brocque 2022). In a medical encounter, human rights should always be bilateral and equivalent, not unilateral (Gross et al. 2018; Tsulukidze et al. 2015).

### 4.5 Limitations of the Work

This study has several limitations. The inclusion of a substantial proportion of participants (33.6%) who were uncertain about being covertly recorded may affect the precision of the results. The regional focus on Western China also limits the generalizability of the findings. Furthermore, the self‐designed questionnaire, although demonstrating good content validity, was not piloted to establish its reliability or structural validity. Additionally, the sample was drawn exclusively from secondary and tertiary hospitals, and thus the findings may not extend to primary care settings where nurse–patient dynamics differ. Finally, the use of a single self‐administered survey without qualitative interviews limits the depth of understanding of the reported experiences.

## 5 Conclusion

In the age of highly developed self‐media, patient‐led covert recording is a common yet under‐addressed source of workplace stress for nurses. This study identifies it as a potential form of workplace violence, strongly opposed by nurses. Therefore, targeted interventions are needed. Hospital managers should prioritise developing clear anti‐recording policies, especially for high‐contact areas like outpatient clinics, and provide corresponding staff training. These steps are essential to mitigate psychological harm to nurses and clarify boundaries for patients. Furthermore, policymakers should address the legislative gaps surrounding covert recording in healthcare. Future research should extend into primary care settings and employ mixed‐method designs to advance understanding while also incorporating pilot testing to establish psychometrically robust measures for this evolving field.

## Author Contributions

**Lanlan Zheng:** methodology, formal analysis, data curation, writing – original draft. **Hui Yang:** conceptualization, investigation, methodology, writing – review and editing, supervision, project administration, validation, resources. **Jie Tang**, **Fang Zhu**, **Jun Yang** and **Feng Peng:** conceptualization, investigation, resources, supervision.

## Funding

The authors have nothing to report.

## Disclosure

Statistic Statement: The statistics were checked prior to submission by the author Hui Yang.

## Ethics Statement

This study was approved by the Ethics Committee for Medical Research and New Medical Technology of Sichuan Cancer Hospital (SCCHEC‐02‐2021‐084) before the data were collected.

## Conflicts of Interest

The authors declare no conflicts of interest.

## Data Availability

The datasets generated during and/or analysed during the current study are available from the corresponding author upon reasonable request.
